# Clarification of the cut-off score for Zung’s self-rating depression scale

**DOI:** 10.1186/s12888-019-2161-0

**Published:** 2019-06-11

**Authors:** Debra A. Dunstan, Ned Scott

**Affiliations:** School of Psychology and Behavioural Science, University of New England, Armidale, NSW 2351 Australia

**Keywords:** Depression screening, Zung self-rating depression scale (SDS), Cut-off score

## Abstract

**Background:**

Zung’s Self-rating Depression Scale (SDS) is an established norm-referenced screening measure used to identify the presence of depressive disorders in adults. Despite widespread usage, issues exist concerning the recommended cut-off score for a positive diagnosis. First, confusion arising from the conversion of raw scores to index scores had resulted in a considerably higher cut-off score than that recommended being used by many researchers. Second, research in China [Chin J Nervous Mental Dis. 12:267-268; 2009] and Australia [BMC Psychiatry. 17:329; 2017] had suggested that the current recommended cut-off is lower than ideal, at least in those countries.

**Method:**

To explore these matters further, sensitivity and specificity figures for alternative cut-off points were examined in positive clinical and negative community samples respectively. The positive clinical sample (*n* = 57) consisted of adults receiving treatment from a medical professional for some kind of depressive disorder, whose diagnosis was positively confirmed using the Patient Health Questionnaire (PHQ). The negative community sample (*n* = 172) was derived from a representative sample of adults whose absence of any depressive disorder was similarly confirmed by the PHQ.

**Results:**

Mathematical models, including Youden’s Index and the Receiver Operating Characteristics Curve, suggest that the recommended cut-off (a raw score of 40) is indeed too low. More detailed comparisons, including consideration of the likely numbers of false positives and negatives given prevalence rates, confirm that, ironically, the incorrect SDS cut-off score mistakenly applied by many researchers (a raw score of 50) would appear to provide far greater accuracy.

**Conclusions:**

Research in China [Chin J Nervous Mental Dis. 12:267-268; 2009] has resulted in an elevated SDS cut-off score of 42 being used in many Chinese studies. Research by Dunstan and Scott [BMC Psychiatry. 17:329; 2017] in an Australian context, suggested that a greater increase, to a raw score of 44 might be required. Based on this study, an even larger adjustment is required. Specifically, we recommend the use of an SDS raw score of 50 as the cut-off point for clinical significance.

## Background

Diagnosing mental disorders in accordance with the Diagnostic and Statistical Manual of Mental Disorders (DSM; [1]) strictly requires a clinical interview but this process is both expensive and time-consuming. As a result, multiple psychometric questionnaires have been developed for use in screening for different conditions. Such screeners can be either criterion-referenced or norm-referenced. The former, of which the Patient Health Questionnaire (PHQ; [2]) is a prominent example, directly address the DSM criteria for the disorder in question. The latter provide each individual with a score which reflects the extent they report experiencing difficulties associated with the condition. Cut-off points are set by comparing the scores of norm-refernced groups with and without the disorder. Participants whose score equals or exceeds the cut-off are considered likely to be sufferers [3].

The Self-rating Depression Scale (SDS) developed by Zung [4, 5] is a norm-referenced measure, used to screen adults for the potential presence of depressive disorders.

The scale enjoys widespread usage, particularly in the research context. However, questions have been raised regarding both the appropriateness and the correct application of cut-off scores. The scale produces raw scores between 20 and 80, however Zung [4] recommended converting these to Index Scores (which ranged between 25 and 100) by the simple process of multiplying by 1.25. Zung’s recommended cut-off for identifying adults with depressive disorder was index scores of 50 and over. Dunstan and Scott [6] identified that many researchers were mistakenly applying this 50 point cut-off to raw scores rather than index scores. Where this occurred, multiple partcipants who should, at least technically, have been classified as suffering from depressive disorders were not so identified. However, it has also been suggested that Zung’s recommended cut-off may have been set at too low a level (at least for populations outside the US). Wang, Cai, and Xu [7] suggested that an index score of 53 (raw score 42) was more appropriate for use with Chinese populations and this suggestion has since been adopted in a number of Chinese studies e.g., [8, 9]. Similarly, research by Dunstan, Scott, and Todd [10] suggested that an index score of 55 (raw score 44) might be more appropriate for use in an Australian context. This study further examines the question of what constitutes the appropriate cut-off score for the SDS. To avoid any further confusion between raw and index scores only raw scores will be referred to from this point onwards.

### Methods for setting cut-off scores

Mathematical approaches to the setting of cut-off scores include the Mean ± 2SD method, the Youden Index, and use of the Receiver Operating Characteristics (ROC) curve [3, 11].

The Mean ± 2SD method effectively serves to identify the cut-off points that would be expected to yield 95% specificity and 95% sensitivity respectively. For a scale which is designed such that higher scores are more indicative of a positive diagnosis these points are calculated as follows: looking first within the non-clinical sample, the Mean Score + 2 Standard Deviations (SD) represents 95% specificity; similarly, within the clinical sample, the Mean Score – 2 SD represents 95% sensitivity. These points can be considered to provide the limits for the choice of a cut-off point. Assuming, as will normally be the case, that the Mean + 2SD for the non-clinical sample is greater than the Mean – 2SD for the clinical sample, then the point of intersection of the two normal curves offers a natural point of compromise [3].

The Youden Index for any potential cut-off score is defined as the sum of the sensitivity and specificity (expressed as probabilities) of the scale at that point minus 1. The cut-off is selected as the point with the highest Youden Index. It is worth noting that this method effectively treats sensitivity and specificity as being of equal importance: false positives and false negatives are equally undesirable [12, 13].

**Fig. 1:**
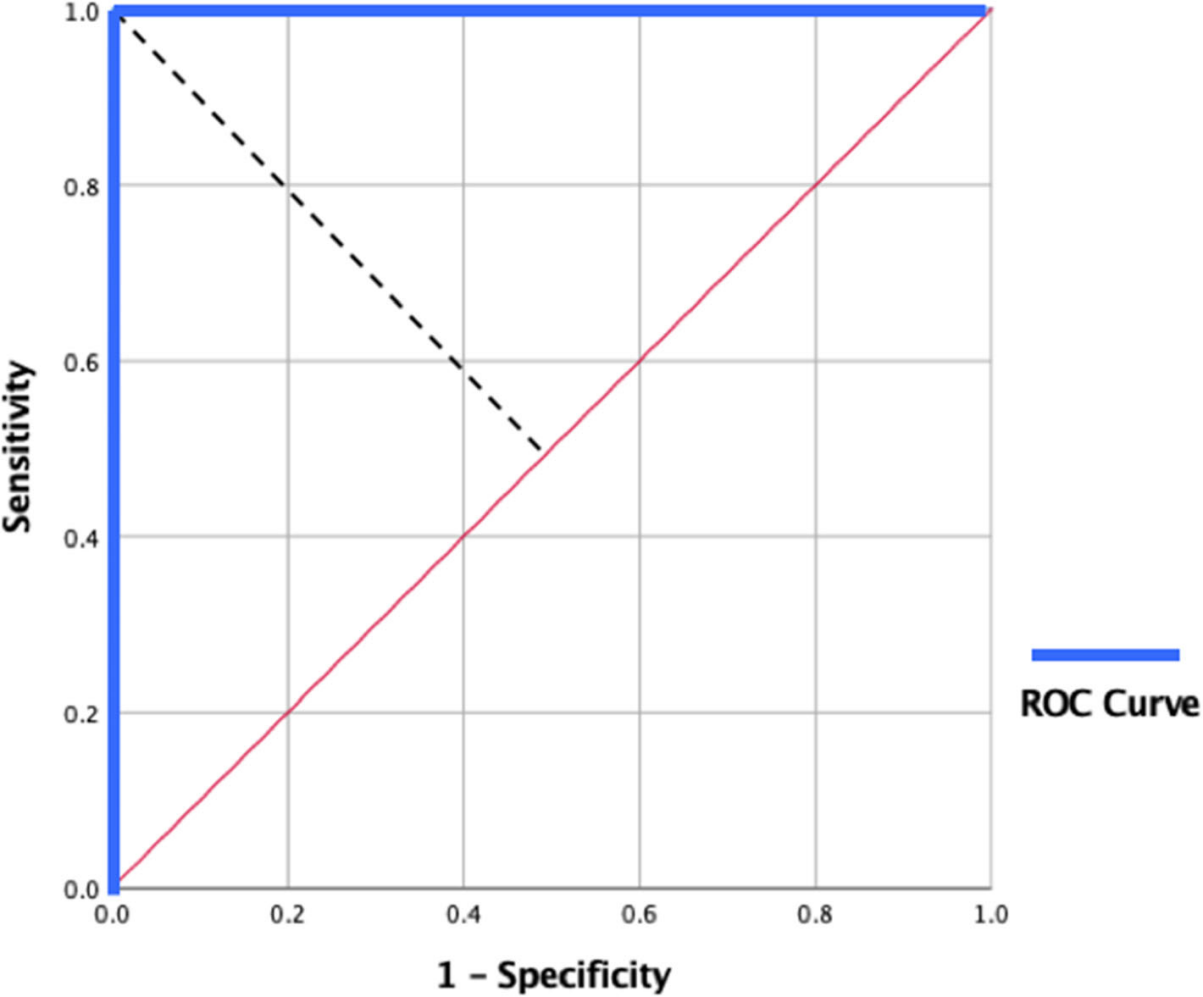
The ROC curve (blue line) for a perfectly discriminating test. Whatever cut-off point is chosen, either sensitivity or specificity = 1, hence the ROC curve in such a case consists of two straight lines. The dotted black line represents the points where sensitivity and specificity are equal

The ROC curve plots sensitivity (on the y-axis) versus 1 – specificity (on the x-axis) for all possible cut-off points. For a test capable of perfectly identifying positive and negative diagnoses, whatever possible cut-off point is chosen, either sensitivity or specificity (or both) will have a value of 1. In this case, therefore the ROC ‘curve’ is actually two straight lines, one along the y-axis from the origin to the point where y = 1, and the other parallel to the x-axis from this point to the point at which x = 1 (as shown in Fig. 1). The intersection of these lines, the point (0, 1), represents the cut-off point(s) which perfectly identify the correct diagnoses, that is where sensitivity and specificity are both 1. One ROC curve method is to set the cut-off point as the value at which the distance from this perfect point is minimal [14]. This method tends to provide a better balance between sensitivity and specificity. However, if this balance is considered to be of overriding importance, a further alternative is to set the cut-off to correspond to the point where the curve intersects the line representing the points where sensitivity and specificity are equal (Fig. 1; [11]). In addition, the area under the ROC curve provides a measure of the test’s ability to correctly discriminate between subjects with and without the disorder concerned: the greater the area, the more discriminating the test [14].

While it should be acknowledged that the use of the above methods can lead to inflated estimates of sensitivity and specificity, particularly where smaller samples are involved [11, 15], they provide valuable context for assessing the merits of alternative cut-off candidates.

### Zung’s recommended cut-off score

Zung’s [16] first mention of a cut-off score for the SDS comes in his paper entitled “How normal is depression”. Raw scores of 40 and above are considered indicative of the presence of depression. The criteria for selecting this point are not specified but Zung quotes both means and standard deviations obtained for ‘normal’ and clinical populations and also provides measures of sensitivity and specificity. The prime focus is on 20 to 64 year-olds. Here the cut-off score represents the Mean + 1.2 SD for the ‘normal’ sample and the Mean – 1.2 SD for the clinical sample. Amongst this age group, sensitivity and specificity measures for the cut-off selected are both 88%. However, Zung [16] also reports specificity measures of 52 and 56% for under 19 year-olds and those aged 65 and over.

### The current study

This study further explores what would constitute an appropriate cut-off score for the SDS in a modern Australian context. Crucially unlike the Dunstan et al. study [10], whose findings were somewhat compromised by the nature of the samples used, the samples used here were representative of the adult population.

## Method

### Participants

The study involved both clinical and community samples. The clinical sample consisted of 148 adults (54 men and 94 women; mean age 46.94 years [SD = 15.19, range = 18–83]) who identified as receiving treatment from a mental health professional for depression. The community sample consisted of 210 adult participants (108 men and 102 women; mean age 45.59 years [SD = 17.43, range = 18–82]). Participants were recruited from Qualtrics survey panels with exclusion criteria to eliminate individuals who were unable to read and understand English, who had suffered a major loss in the last six months, or who had been diagnosed with a mental illness involving psychotic features. Additionally, the community sample excluded individuals who qualified for the clinical sample or who were receiving treatment from a mental health professional for an anxiety disorder.

### Procedure

Qualtrics survey panel members meeting the sample criteria were invited to complete an online survey taking approximately 10 min. Completion and submission of the survey was entirely voluntary.

### Measures

In addition to the collection of demographic and biographical information the survey involved completion of the following scales:

- Zung Self-rating Depression Scale (SDS)
  - The Zung SDS consists of 20 self-report items that were identified in factor analytic studies of the syndrome of depression [4]. Items tap psychological and physiological symptoms; 10 express negative experience such as “I feel down-hearted and blue” and 10 express positive experience and are reverse scored such as “I eat as much as I used to”. Respondents rate each item according to how it applied to them within the past week using a 4-point scale ranging from 1 (*none, or a little of the time*) to 4 (*most, or all of the time*). Total raw scores range from 20 to 80. The SDS has fair internal consistency, with a split-half reliability of .73. An alpha coefficient of .68 was reported by Deforge and Sobal [17] while other authors have reported .79 [18] and .81 [19]. Reported correlations with other depression scales include .41 with the Hamilton Rating Scale [20], .54 with the Depression Adjective Checklist, and .68 with the Beck Depression Inventory [19]. Cronbach’s alpha for the scale on the current study was .87.
- Patient Health Questionnaire (PHQ)
  - The PHQ is designed as a brief, user-friendly self-report measure with items corresponding to a range of DSM-IV diagnostic criteria. It should be noted that all the DSM-IV criteria on which the PHQ is based remain unchanged in the current edition of the manual, DSM-5 [1, 21]. This study utilised the two-page version, covering Major Depressive Disorder and Other Depressive Disorder (9 items), and Panic Disorder and Other Anxiety Disorder (22 items). Compared with diagnoses made by mental health professionals, the sensitivity of the PHQ in relation to depressive disorders is 61% and the specificity 94% [2].

## Results

Table 1 details the number of participants in both the clinical and community samples meeting the PHQ criteria for Depressive Disorders. A total of 38.5% of the clinical sample and 18.1% of the community sample met the PHQ criteria for some form of depressive disorder.

**Table 1:** Number of PHQ depressive disorder diagnoses by sample

	Clinical sample (*n* = 148)	Community sample (*n* = 210)
Major Depressive Disorder	46	28
Other Depressive Disorder	11	10
Total Depressive Disorder Diagnoses	57	38

Both clinical and community samples were further divided into subsamples based on whether or not participants screened positive for some form of depressive disorder on the PHQ. Table 2 details the mean SDS scores for these four sub-samples. Within both the clinical and community sample, an independent samples *t*-test confirmed that, as would be expected, those receiving a positive diagnosis for depressive disorders on the PHQ registered significantly higher scores on the SDS. For the clinical sample, *t*(142.2) = 8.65, *p* < .001; for the community sample, *t*(208) = 9.75, *p* < .001.

**Table 2:** Mean SDS scores for participants screening positive and negative for Depressive Disorders on the PHQ

SDS mean score (SD)	Clinical sample	Community sample
PHQ Positive Depressive Diagnosis	54.98 (7.25)	53.92 (6.91)
No PHQ Diagnosis	42.77 (9.87)	38.10 (9.45)

Before examining sensitivity and specificity figures within these subsamples, certain observations are necessary. First, members of the clinical sample cannot be assumed to still be experiencing symptoms of depression. Although all professed to be receiving treatment, in an unspecified number of cases that treatment (which could be either pharmaceutical or psychotherapeutic in nature) can be expected to have induced a sufficient reduction in symptoms as to render a positive diagnosis no longer appropriate. Our identification of sufferers of depression is therefore dependent on the PHQ, but the PHQ is itself less than perfect. Table 5 details the expected number of false positives and negatives within each sample on the basis of the sensitivity (61%) and specificity (94%) figures reported by Spitzer et al. [2]. While the actual numbers undoubtedly will differ somewhat, these figures constitute our best guess.

Examination of Table 3 reveals that while false diagnoses are likely to be relatively rare in the Positive Clinical and Negative Community subsamples (approximately 6–7% and 10–11% respectively), they are of a magnitude likely to severely compromise the integrity of the Positive Community and Negative Community samples. Hence, in examining sensitivity and specificity figures for the SDS, only those figures relating to the Positive Clinical sample and the Negative Community sample have been considered (Tables 4 and 5). This effectively mirrors the approach taken by Zung [16].

**Table 3:** Approximate numbers of false positives and negatives anticipated in PHQ diagnoses by sample

PHQ Diagnosis/Original sample		
	True Positives	False Positives
Positive Clinical (*n* = 57)	53–54	3–4
Positive Community (*n* = 38)	28	10
	True Negatives	False Negatives
Negative Clinical (*n* = 91)	57	34
Negative Community (*n* = 172)	154	18

**Table 4:** Positive Clinical Sample (*n* = 57): Percentage of cases also diagnosed as positive on the SDS by cut-off point

Cut-off point	Sensitivity
38	100.0
39	100.0
40	98.2
41	98.2
42	96.5
43	96.5
44	93.0
45	93.0
46	91.2
47	91.2
48	87.7
49	82.5
50	78.9
51	74.7
52	64.9

**Table 5:** Negative Community Sample (*n* = 172): Percentage of cases with that would also receive no diagnosis on the SDS analysed by cut-off point

Cut-off point	Community sample (*n =* 172)
38	50.6
39	53.5
40	57.0
41	58.7
42	59.3
43	62.8
44	67.4
45	70.9
46	72.1
47	74.4
48	76.7
49	82.0
50	83.7
51	93.6
52	95.3

Utilising these two samples, the Mean ± 2SD method sets the lower value for the cut-off point (derived from the Negative Community Sample) at 40.4 and the upper value (from the Positive Clinical Sample) at 57.1. The point at which the two normal curves cross is 47.6.

Turning now to the Youden Index, its maximum value of .683 is achieved by setting the cut-off score for a positive SDS diagnosis at a raw score of 51; at this score sensitivity is 75% and specificity 94%. In contrast, the Youden Index for the existing cut-off of 40 is .552 and for the cut-off of 44 recommended by Dunstan et al. [10] is .604.

**Fig. 2:**
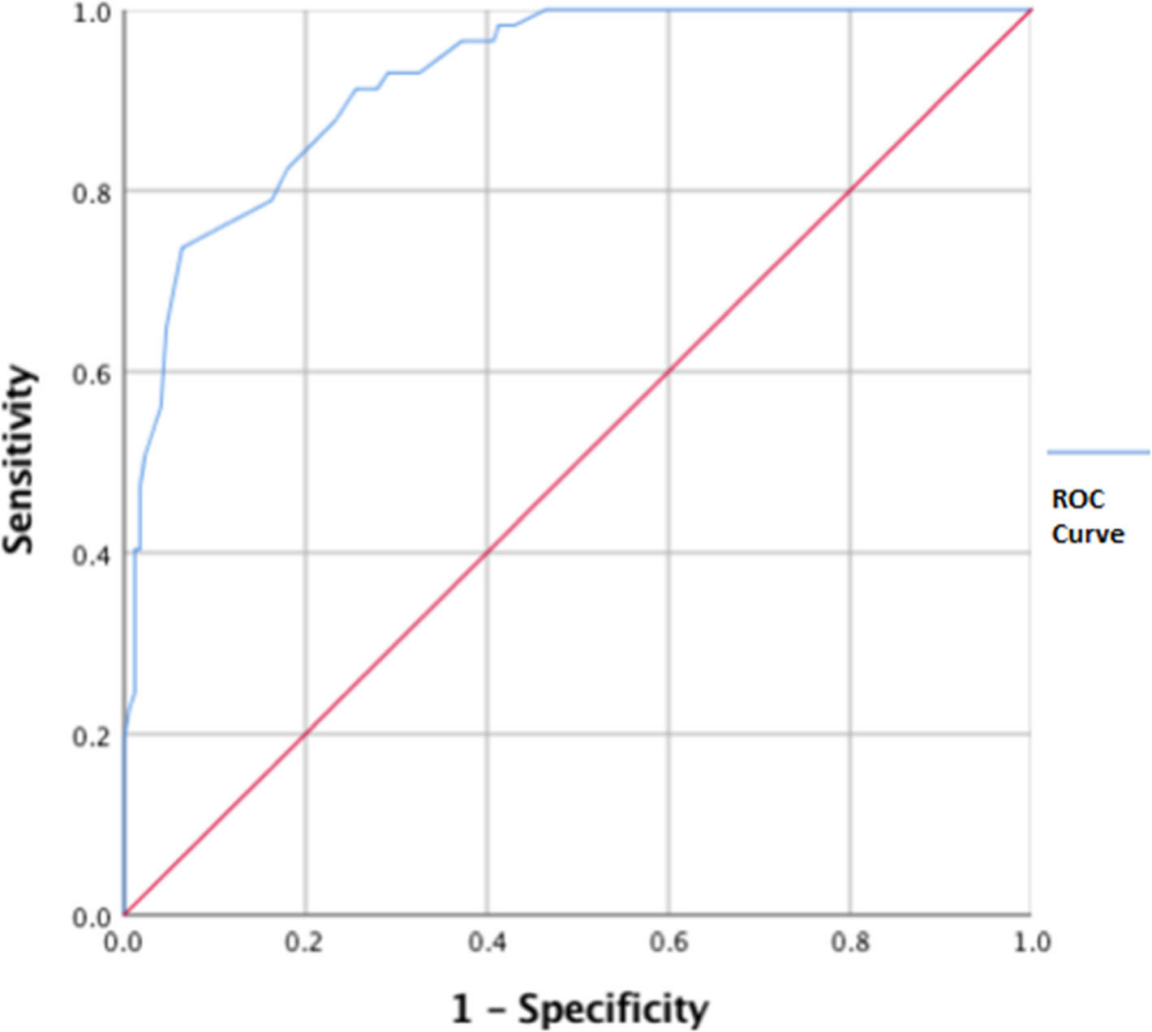
The ROC curve (blue line) obtained for the combination of the Positive Clinical and Negative Community samples. For each possible SDS cut-off point, the sensitivity of the SDS amongst the Positive Clinical subsample is graphed against 1- the specificity recorded amongst the Negative Community subsample

The ROC curve that results from this combination of the Positive Clinical and Negative Community samples is shown in Fig. 2. The closest point on the curve to the top-left corner of the graph, at a distance of .251, occurs with the cut-off set at 49. This point also represents the best balance between sensitivity and specificity (83 and 82% respectively). In contrast, the point corresponding to the current 40 cut-off is at a distance of .430 and that corresponding to 44 is at a distance of .333. The area under the curve equals .92 (95% Confidence Interval: .89, .96).

## Discussion

Reviewing these results, it is clear that mathematical methods suggest that the cut-off score of 40 for the SDS should not only be increased but increased beyond the score of 44 suggested by Dunstan et al. [10]. Optimal figures from the different mathematical models vary between 48 (Mean ± 2SD method) and 51 (Youden Index). Indeed, ironically, the cut-off score of 50 mistakenly applied by many researchers [6] would appear more appropriate than Zung’s actual recommendation (see Table 6 for an explicit mathematical comparison).

**Table 6:** Comparison of mathematical indices for the current SDS cut-off of 40 and the cut-off of 50 mistakenly applied by many researchers

	40	50
Mean ± 2SD method: cut-off expressed in these terms for:		
Negative Community Sample	Mean + 0.20 SD	Mean + 1.26 SD
Positive Clinical Sample	Mean – 2.07 SD	Mean – 0.69 SD
Youden Index	.552	.626
ROC curve distance from optimum point	.430	.267
Sensitivity	98.2%	78.9%
Specificity	57.0%	83.7%

While these mathematical models offer valuable insight, they are limited in failing to make any allowance for the relative costs attached to false negatives and false positives. Similarly they take no account of the prevalence of the disorder in the population. If the cut-off value chosen is to maximise the benefit that occurs from testing, these are all factors that need to be taken into consideration [22].

The 2007 National Survey of Mental Health and Wellbeing reports the 12-month prevalance of affective disorders in the Australian adult population as being 6.2% [23]. Given a prevalance for depressive disorders of this ilk, then it is possible to estimate the numbers of false positives and false negatives that would occur using the alternative cut-offs under consideration (Table 7).

**Table 7:** Approximate numbers of false positives and false negatives per 100 cases to be expected using SDS cut-offs of 40 and 50

Cut-off	False Negatives	False Positives	Total Misdiagnoses
40	0	40	40
50	1	15	16

As can be seen from Table 7, although the cost of increasing the cut-off to 50 would be to reduce the sensitivity to 78.9%, meaning approximately 1 in 5 sufferers would not be identified, the benefit is a major reduction in the number of false positives to be expected and, hence, a considerable improvement in overall accuracy. Again, reactions to these figures may differ according to the context in which the test is being applied [22]. In a clinical screening context, failing to identify 1 in 5 sufferers may be considered unacceptable. However, even here, the figures suggest some increase from the current cut-off would be advisable so as to limit the number of false positives. In a research context, where false positives and negatives are equally undesirable, it is ironic to note that those researchers who mistakenly applied the incorrect cut-off score of 50 would seem likely to have achieved greater accuracy in their classifications.

## Conclusions

In sum, the potential value of the SDS as a screener for clinically significant depression is evidenced by the above results, including the high value registered for the area under the ROC curve and sensitivity and specificity figures which compare favourably with those reported for similar indices such as the Depression subscale of Lovibond and Lovibond’s [24] Depression Anxiety Stress Subscale e.g., [10, 25, 26]. Based on our findings, we recommend the use of an SDS raw score of 50 as the cut-off point for clinical significance.

## Data Availability

The study data is available from the corresponding author on application.

## Abbreviations

DASS: Depression Anxiety Stress Scale
DSM: Diagnostic and Statistical Manual of Mental Disorders
PHQ: Patient Health Questionnaire
ROC: Receiver Operating Characteristics
SDS: Zung Self Rating Depression Scale
